# West Country Physicians Club

**Published:** 1986-04

**Authors:** 


### West Country Physicians Club

**Seventy seventh Meeting held at Exeter on October 25th and 26th, 1985**
Abstracts of papers

### DEATHS IN ANGUILLA 1885–1984

**C. J. BURNS-COX**
**Frenchay Hospital, Bristol**

Anguilla is a British island of about 6,500 people on 35 square miles. It lies at the North East tip of the Lesser Antilles 18° North.

The age structure of the population has changed recently, for example in 1966 46% were less than 15 and 8% over 64 whereas in 1984 the figures were 35% and 11%.

Birth and death registers are complete from 1883 and I have analysed them by quinquennia.

The infant mortality rate has fallen from 114 in 1945, at about which time the first community nurse was employed, to 32 in the last 5 years. Deaths under 5 as a percentage of total deaths have fallen over the same period from 32% to 9%. 54% of deaths now occur over the age of 69.

Childbirth, neonatal tetanus, measles, diarrhoea and tuberculosis have disappeared as significant causes of death and if a child survives the first 7 days of life he is exceedingly unlikely to die before 5.

There have been marked increases in deaths from stroke, myocardial infarcts and cancer. The latter occurs chiefly in stomach, prostate, oesophagus, colon and lung in men and uterus, stomach, breast, colon and ovary in women.

Anguilla used to be very poor and is still far from wealthy, nevertheless the change in mortality patterns deserve admiration. I believe that the work of community nurses and the disappearance of undernourishment are the chief factors in such improvements.

I thank the Government of Anguilla and the Overseas Development Administration, London for permission to make this study.

### FACTORS AFFECTING MEASUREMENT OF GLYCOSYLATED HAEMOGLOBIN

**M. Hartog**
**Southmead Hospital, Bristol**

Non enzymatic glycosylation of proteins is a universal phenomenon the extent of which is determined by the rate of turnover of the protein and the prevailing level of blood glucose. Glycosylated haemoglobin (Hb) is widely used as an integrated index of diabetic control over the previous 6–10 weeks. It is most commonly measured either by a chemical technique or by a physical method of separation, such as electrophoresis, which depends on the difference in mobility between glycosylated Hb ('fast' haemoglobin) and HbA.

The correlation between results obtained by the two methods is usually good. Physical separation methods, however, are affected by haemoglobinopathies in particular by persistence of HbF which is indistinguishable on electrophoresis from glycosylated Hb. Adducts other than glucose such as acetaldehyde (from alcohol), acetyl group (from acetyl salicylic acid) and carbamylation (from cyanate derived from urea in uraemia) may also affect physical separation methods. Thus in a study of non diabetic and diabetic uraemics we have found that glycosylated Hb is significantly higher when measured by electrophoresis than chemically in subjects with very high blood urea levels, presumably because of the presence of carbamylated Hb.

These factors have to be remembered in assessing the significance of measurement of glycosylated Hb by physical separation methods.

### RATIONAL PRESCRIBING FOR DUODENAL ULCER

**J. G. Williams**
**Surgeon Commander, Royal Naval Hospital, Haslar, Gosport, Hants**

It has been recognised for many years that some patients with duodenal ulcer disease display increased basal and nocturnal acid secretion. The night is the longest single period of unbuffered intragastric acidity and studies with duodenal ulcer patients have shown that inhibition of intragastric acidity over 24 hours is similar with either cimetidine 400 mg bd or 800 mg at night. In clinical trials similar healing rates have been obtained with these two doses, with one trial showing significantly better healing rates with the nocturnal dose. Review of healing rates in a large number of published trials has shown that the most effective regimens for $H_2$-antagonists use a potent single nocturnal dose (cimetidine 800 mg or ranitidine 300 mg) given for eight weeks. Such treatment will achieve over 95% healing.

These doses given at 2300 hours cause effective inhibition of overnight acidity with little effect the next morning. Earlier dosing (at 1800 hours) causes longer but less profound inhibition and would be unlikely to improve healing rates. Higher doses of cimetidine (1200 or 1600 mg) will cause anacidity overnight in most subjects. Such doses have not been evaluated in clinical trials.

A single nocturnal dose of $H_2$-antagonist, given on retiring is now established as a most effective treatment for duodenal ulceration.

### ISCHAEMIA AND SINUS NODE DISEASE

**D. B. Shaw**
**Royal Devon and Exeter Hospital, Exeter EX2 5DW**

The aetiology of chronic sinoatrial disorder (sick sinus syndrome) has been a matter of dispute for a number of years. At first it was assumed to be the result of ischaemic heart disease. Subsequently pathological studies of several small series failed to show evidence of gross coronary disease. However full angiographic study has not been described nor has the question of a 'steal phenomenen' been investigated.

The paper described postmortem angiography in a series of patients with chronic sinus node disease and compared the findings with those obtained in subjects dying with chronic atrioventricular block but no evidence of sinus node dysfunction. It was concluded that coronary artery disease was unlikely to be the aetiology of sinus node dysfunction in a majority of patients studied, but some impairment of blood supply was seen in almost a third of cases. A further series of four patients with acute disturbance of sinus node function following myocardial infarction was described. Two of these survived for over a fortnight. It was suggested that future improvement in the outlook of patients with myocardial infarction might lead to an increase in the numbers of patients surviving with a chronically ischaemic sinus node.

### INTESTINAL BLOOD FLOW AND ABSORPTION

**R. H. Mountford**
**Bristol Royal Infirmary, Bristol**

Modern ultrasonic scanners permit the visualization of the superior mesenteric artery and determination of its diameter. Pulsed Doppler flow-meters allow measurement of blood flow velocity within such a vessel. From these parameters, total superior mesenteric artery blood flow (SMABF) can be calculated. This method is reproducible and yields results similar to those obtained by more invasive methods.

Using this method, SMABF has been shown to increase from the fasting state by some 60% following ingestion of both solid and liquid meals. The time course of this change varies according to the composition of the test meal.

The mechanism by which this post-prandial increase in blood flow occurs is unknown. This was investigated in nine healthy volunteers, each examined on two separate occasions. At each experiment, they were given an identical volume (400 ml) of isotonic solution to drink. However, on one occasion this contained a soluble sugar (glucose) and on the other this contained a non-absorbable carbohydrate (Lactulose).

The expected prompt rise in SMABF occurred in all subjects following the ingestion of glucose. However, blood flow remained at basal levels following lactulose.

This observation suggests that absorption of food substrate play an important role in controlling post-prandial increase in blood flow in the superior mesenteric artery.

### ROC CURVES: AIDS TO DIAGNOSIS

**G. H. Hall**
**Royal Devon and Exeter Hospital, Wonford, Exeter**

Usually, a test result is classified as abnormal if it lies outside the normal 'reference range'. However, patients with disease may have normal results (false negatives) and normal people may have abnormal results (false positives). Proper interpretation of a result requires information about the degree of overlap of the normal and diseased distributions of test results. Different cut-off points will alter the ratio of true positive to false positive results. This relationship is displayed in the ROC (Receiver Operating Characteristic) curve. The value of such a curve in the evaluation of antibody tests for AIDS was demonstrated.

### DUAL CHAMBER PACEMAKERS— PROBLEMS AND COMPLICATIONS— A REVIEW OF 70 PATIENTS

**J. E. Sanderson**
**Musgrove Park Hospital, Taunton**

Dual chamber (A. V. sequential) pacing is a significant advance in pacemaker techology. The introduction of atrial electrodes which are stable in the right atrial appendage and advances in pacemaker electronics and circuitry have been a major factor in this development. This form of pacing is the most logical solution to complete heartblock at the moment and since it is not difficult to implant two electrodes instead of one we have implanted dual chamber pacemakers in all suitable patients with complete heartblock and some selected cases of sick sinus syndrome.

During the period July 1981 to March 1985 we implanted DDD pacemaker units into 70 patients (41 men, 29 women—age range 45–84 years). The electrodes were introduced predominantly by the direct subclavian puncture technique. After implantation the pacemakers were programmed to produce the optimum readings and all patients were tested for myopotential inhibition by standard testing (pectoral muscle stimulation) and in 38 patients twenty-four hour ECG recordings have also been done.

The general complications were few; angina (1 patient) which was abolished by reprogramming the pacemaker, wound infection (2 patients) cleared with antibiotic treatment, DVT (1 patient), pneumothorax (1 patient), haemorrhage from subclavian vein (1 patient) and severe haematoma formation (1 patient). In 7 patients the atrial electrode displaced and required repositioning but this has become much less of a problem in the last few years. Loss of atrial sensing occurred in 2 patients and the pacemakers were reprogrammed to DVI mode. Displacement of the ventricular lead occurred in only 2 patients. A rise in ventricular pressure occurred in another 2 patients and this was abolished by reprogramming the pacemaker. A few patients developed supraventricular arrhythmias (atrial tachycardia—3 patients, atrial flutter—2 patients, atrial fibrillation—2 patients). These were all successfully treated with drugs or by reprogramming the pacemaker to DVI mode. 3 patients developed a pacemaker mediated tachycardia, which was precipitated by myopotential sensing in all but which was abolished by reprogramming the pacemaker to prolong the atrial refractory period.

Myopotential sensing was looked for in all patients. We found that ventricular inhibition could be demonstrated in 63% of the patients but it appeared to be responsible for symptoms in only 15%. Atrial myopotential sensing was much less common (16%) but caused symptoms in nearly half the patients (42%). Only 1 patient remained persistently symptomatic from myopotential sensing.

Several patients in this group have died but all deaths were unrelated to the pacemaker. Symptomatic improvement was considerable. 24 patients were assessed as having an excellent result, 42 patients a good result and 4 patients a fair result. 11 patients who were converted from a VVI system to DDD pacing all volunteered that they felt considerably better.

This study has shown that DDD pacing can be done with a low complication rate almost comparable to single chamber pacing in a district general hospital. The advantage is that there appears to be considerable improvement of the patient's symptoms with DDD pacing and it would now appear to be the treatment of choice for complete heartblock.

